# Letter from Professor Martins

**Published:** 1840-07-18

**Authors:** 


### MEDICAL EXAMINER.

DEVOTED TO MEDICINE, SURGERY, AND THE COLLATERAL SCIENCES.

No. 29.] PHILADELPHIA, SATURDAY, JULY 18, 1840. [Vol. III.

### FOREIGN CORRESPONDENCE.

### LETTER FROM PROFESSOR MARTINS.

No. IX.

*Course of Therapeutics at the Faculty of Medicine—M. Trousseau on Potassa, and some of its Combinations.*

Paris, 1*st June*, 1840.

To the Editors of the Medical Examiner.

The potassa employed in medicine is a hydrate of the protoxide of potassium. It is principally used for the purpose of cauterization. The details into which we are about to enter, are worth the notice of practitioners: sufficient attention is not paid to them, and yet every day the physician is liable to be questioned upon the application or management of an issue. To effect cauterization, we may employ, 1st, alcoholic potassa; 2dly, potassa with lime; 3dly, the Vienna powder.

Alcoholic potassa, or the pure hydrate, acts with great energy. It forms a deep eschar; but it is liable to the great disadvantages of absorbing rapidly the surrounding moisture, of running, and producing irregular, and often very extensive eschars. It must not be forgotten that the eschar is usually eight or ten times the diameter of the piece of potash employed, and that it runs sometimes to a considerable distance under the porte-caustic. The potassa with lime should be preferred, as it is much less deliquescent, produces a dry eschar, the diameter of which is but four or five times that of the piece of potash employed. But of all these preparations, the best is unquestionably the Vienna powder. It is composed of an intimate mixture of equal parts of alcoholic potassa and quicklime, which are triturated in a hot iron mortar, and afterwards preserved in a well-stopped bottle. This powder produces an eschar of a diameter equal to that of the substance employed. It is to be applied in the following manner. A certain quantity, say a half a drachm, of this powder, is triturated with alcohol, or cologne water, or any other alcoholic liquid. There results a paste, or rather a mortar, which is placed either in a tube, the bottom of which is filled with cotton, or on a plate of lead or a piece of oil-cloth, pierced with a hole exactly of the size of the eschar desired. It is applied for ten minutes, and the eschar results. Fifteen minutes are rarely necessary to produce it. The period at which the eschar separates is very variable. Never in less than ten days, sometimes a month is necessary; the average period is from fifteen to twenty days. In some subjects, it does not detach itself at all; that is to say, it consumes itself, becomes reduced to the thinness of letter paper, and when it peels off, the cicatrix is formed beneath. This is a phenomenon which is particularly observed in thin subjects. In general, the eschar detaches itself so much the more easily, as the vitality of the skin is the more active. But it is not necessary to wait till it falls off, to insert the issue pea. It may be introduced at the end of six days, and sometimes before, if the eschar is soft in the centre, as is the case when the potash has penetrated deeply. It is by no means an indifferent matter of what substance is the pea inserted in the hole. Common peas, the grains of the *Pisum sativum*, are liable to the inconvenience of swelling considerably, often unequally; they thus enlarge the issue, and cause the patient great pain. They should not, therefore, be used, except when it is intended to enlarge the cavity. The peas of the box-tree, (*Buxus sempervirens*,) or of the guyacum, (*Guyacum officinale*,) do not swell, and hence do not irritate the wound. The same results take place with the unripe fruit of the orange tree, or with caoutchouc, which has lately been employed. Issues made with the root of the Florentine orris, (*Iris Florentina*,) have an agreeable odour, but they contain an acrid, irritating principle, and sometimes give rise to erysipelas or chronic eczemas. They are therefore to be preferred when it is desired to increase the activity of the suppuration, but are to be laid aside when it is well established.

*The Bicarbonate of Potash.*—We propose here to notice the alkaline bicarbonate, or more exactly to speak, of the sesquicarbonate of potassa. This substance is useful in the following affections:

1st. The pyrosis, or water brash, which is developed under the influence of fat. The lactic and muriatic acids being secreted in abnormal quantity, magnesia, or the bicarbonate of potash, is used to neutralize them. The latter salt is the more efficacious.

2dly. Infantile diarrhœa. During the period of dentition, children are often liable to diarrhœas of greenish stools, (from four to six in the twenty-four hours,) mixed with mucosities, and accompanied by vomiting and colicky pains. These derangements of the digestive organs are owing to the presence of too great a quantity of acid in the stomach. No sooner does the milk reach the stomach than it coagulates, and is immediately thrown up by the child. The preparation best adapted to this condition of the stomach, is the sugar of Vichy, which consists of two drachms and a half of bicarbonate of potassa, mixed with eight ounces of double refined sugar pulverized. It is administered to children in a spoonful of water. If it is not retained, from half a drachm to a drachm of the same salt is to be taken by the nurse, by which means the milk becomes more alkaline, and a portion of the acid of the stomach is neutralized. This remedy, sufficiently long persisted in, has saved the life of many an infant, who, as is well known, most usually fall victims to diseases of the intestinal canal.

3dly. In adults, the alkaline bicarbonate is of the same service. When the secretion of the acid is too active during digestion, the unpleasant results occasioned are well known—diarrhœa, and sometimes vomiting. The patient should in this case drink a bottle of Vichy water a day. This, given to a healthy man, would produce constipation in a short time.

4thly. In organic diseases of the stomach, the bicarbonate of potash, although without pretensions to a radical cure, is of great relief. M. Trousseau has seen patients presenting all the rational symptoms of commencing scirrhus (the tumour excepted) become cured under the influence of this remedy.

5thly. Chronic or subacute diseases of the liver, improve under the use of this salt, particularly under the form of the waters of Vichy, Ems, and Carlsbad, which hold it in solution. It is not to be doubted, that if patients went to the country, and took the same dose of the bicarbonate as they would drink in the water of Vichy, they would be cured. At Vichy, from an ounce to an ounce and a half is swallowed in solution, while at home hardly a few drachms are taken.

The pastils of Vichy or Darcet are made with gum tragacanth, and contain each a grain of bicarbonate of potassa—too little to be used as a remedy. But the waters themselves contain about a drachm of the alkaline bicarbonate to the litre of water. Their sulphurous odour is owing to the decomposition of a slight quantity of sulphate of lime. The waters of Carlsbad, in Bohemia, have less of the bicarbonate; but they have, besides, half a drachm of sulphate of soda to the litre, which renders them more purgative. Those of Ems have a half less of the bicarbonate than those of Vichy, but they are impregnated with carbonic acid, which renders them slightly tonic, and more agreeable to the taste.

